# The role of ^18^F-FDG PET-CT in predicting progressive pulmonary fibrosis in idiopathic inflammatory myopathy associated interstitial lung disease

**DOI:** 10.3389/fimmu.2026.1862578

**Published:** 2026-10-29

**Authors:** Chao Sun, Ze Zhang, Qianqian Zhao, Lin Huang, Jiaxin Tian, Wenxin Wu, Yijie Li, Jialin Teng, Hua Cao, Min Zhou

**Affiliations:** 1 Department of General Medicine/Internal Medicine, Ruijin Hospital, Shanghai Jiao Tong University, School of Medicine, Shanghai, China; 2 Shanghai Ninth People’s Hospital, Shanghai Jiao Tong University School of Medicine, Shanghai, China; 3 Department of Pulmonary and Critical Care Medicine, Ruijin Hospital, Shanghai Jiao Tong University, School of Medicine, Shanghai, China; 4 Institute of Respiratory Diseases, Shanghai Jiao Tong University School of Medicine, Shanghai, China; 5 Shanghai Key Laboratory of Emergency Prevention, Diagnosis and Treatment of Respiratory Infectious Diseases, Shanghai, China; 6 Department of Rheumatology and Immunology, Ruijin Hospital, Shanghai Jiao Tong University, School of Medicine, Shanghai, China; 7 Department of Dermatology, Ruijin Hospital, Shanghai Jiao Tong University, School of Medicine, Shanghai, China

**Keywords:** 18F-FDG PET-CT, idiopathic inflammatory myopathy, interstitial lung disease, prognosis, progressive pulmonary fibrosis, risk factors

## Abstract

**Aim:**

This study aims to investigate the role of ^18^F-FDG PET-CT in the development of progressive pulmonary fibrosis (PPF) in patients with idiopathic inflammatory myopathy-associated interstitial lung disease (IIM-ILD) and to develop a predictive nomogram model.

**Methods:**

This study retrospectively included patients diagnosed with IIM-ILD who underwent ^18^F-FDG PET-CT at Ruijin Hospital between 2018 and 2024. The baseline characteristics, IIM types, myositis antibodies, laboratory tests, pulmonary function, ILD subtypes, ^18^F-FDG PET-CT findings, and treatment were collected. Enrolled patients were divided into PPF and non-PPF groups according to diagnostic criteria. Clinical features were compared between the two groups, and logistic regression analysis was performed to identify risk factors for PPF. All identified risk factors were combined to construct the predictive nomogram. Receiver operating characteristic curve, calibration plot, and decision curve analysis were used to evaluate the accuracy and efficiency of this predictive model. Additionally, we drew the survival curve of PPF and non-PPF, and compared the maximum standardized uptake value (SUV_max_) between short-term death and long-term survival.

**Results:**

A total of 71 eligible IIM-ILD patients were included, of whom 29 developed PPF within one year of follow-up. Multivariate analysis revealed that bilateral lung SUV_max_ and FVC%pred were associated with the occurrence of PPF. The calibration plot demonstrated excellent agreement between the predicted and actual observed probability of PPF (Hosmer–Lemeshow test: *P* = 0.143). The predictive nomogram achieved an area under the curve of 0.887 (95% *CI*: 0.805–0.969). The SUV_max_ in the lungs was significantly higher in the PPF group than in the non-PPF group (*P* < 0.001). Moreover, lung SUVmax was significantly higher in patients who died within one year than in those who survived for more than five years (*P* = 0.014). In addition, patients with PPF had a significantly lower survival rate than those without PPF (*P* = 0.040).

**Conclusion:**

^18^F-FDG PET-CT and FVC% pred are associated with the occurrence of PPF. The prognosis of PPF is poor, and high SUV_max_ is correlated with short-term mortality. Exhibiting robust discrimination and calibration, this nomogram holds potential for clinical application.

## Introduction

Idiopathic inflammatory myopathy (IIM) is a group of heterogeneous diseases characterized by prominent involvement of proximal limb muscles and chronic inflammation. IIM is classified into dermatomyositis (DM), antisynthetase syndrome (ASS), inclusion body myositis, immune-mediated necrotizing myopathy, and polymyositis (1–3). DM can be further divided into clinically amyopathic dermatomyositis and typical dermatomyositis based on the presence or absence of muscle involvement.

Adult-onset IIM is associated with an increased risk of cancer, particularly within three years before and after the onset of IIM (4). Chest-abdomen-pelvic computed tomography (CT), tumor markers, gynecological ultrasound, and endoscopy are commonly used for cancer screening in IIM. Additionally, ^18^F-FDG PET-CT is increasingly utilized for comprehensive cancer screening in IIM, especially in high-risk populations (5–7).

IIM can involve multiple systems including skin, muscle, joints, heart, and lungs, with interstitial lung disease (ILD) being the most common pulmonary manifestation of IIM. Idiopathic inflammatory myopathy-associated interstitial lung disease (IIM-ILD) is categorized based on chest CT features into nonspecific interstitial pneumonia (NSIP), usual interstitial pneumonia (UIP), and organizing pneumonia (OP), with NSIP being the most common subtype (8). Some IIM-ILD cases progress to progressive pulmonary fibrosis (PPF) despite conventional treatment. IIM-ILD is associated with a high mortality rate and poor prognosis (9, 10). Therefore, effectively identifying IIM-ILD patients who develop PPF and implementing stratified management is particularly important.

Studies have shown that ^18^F-FDG PET-CT not only screens for malignancies associated with IIM but also identifies IIM-ILD (11, 12). In recent years, studies have found that lung uptake on PET/CT was associated with ILD. ^18^F-FDG PET-CT and fibroblast activation protein inhibitor (FAPi) PET/CT have been studied most intensively. Several studies found that lung uptake on ^18^F-FDG PET-CT was significantly higher in systemic sclerosis (SSc)- associated ILD (SSc-ILD) and that higher lung uptake was associated with ILD extent and lower DLCO% (13). Furthermore, SSc-ILD patients with higher baseline ^18^F-FDG uptake were more likely to develop PPF. The same results were also observed after utilizing FAPI PET-CT (14). Previous studies found that baseline lung uptake of ^18^F-FDG PET-CT in RP-ILD patients was significantly higher than in non-RP-ILD patients (12). Some small sample studies have shown that whole-lung FAPI PET-CT uptake in IIM-ILD was significantly higher than in non-ILD-IIM patients and the control group. Patients with progressive ILD in one or two years had higher uptake at baseline compared with stable ILD (15, 16). Currently, few studies have reported the value of ^18^F-FDG PET-CT in predicting the development and prognosis of PPF. Thus, this study aims to explore the value of ^18^F-FDG PET-CT in predicting the occurrence of PPF and to identify relevant risk factors for the development of PPF.

## Methods

### Patients

This study retrospectively enrolled hospitalized patients diagnosed with IIM at Ruijin Hospital, Affiliated with Shanghai Jiao Tong University School of Medicine, between January 2018 and December 2024. In our cohort, patients were admitted for initial diagnostic work-up of suspected IIM; evaluation of active or rapidly progressive ILD; initiation or escalation of immunosuppressive therapy; occurrence of serious infection; and/or management of severe systemic manifestations such as dysphagia, respiratory impairment, or cardiac involvement. The inclusion criteria for this study were: (1) age ≥ 18 years; (2) presence of interstitial lung disease (ILD) with fibrosis on high-resolution chest CT (HRCT); (3) completion of ^18^F-FDG PET-CT during hospitalization. The exclusion criteria were: (1) presence of other connective tissue diseases; (2) presence of malignancy; (3) lack of complete follow-up within one year; (4) evidence of acute infection within 4 weeks before PET-CT or with untreated chronic infection.

### Data

Retrospectively collected data during the patients’ initial hospitalization included basic information, clinical manifestations, types of myositis, myositis antibodies, laboratory tests, imaging examinations, pulmonary function, and treatment. The follow-up data (respiratory symptoms, HRCT, pulmonary function) within one year were evaluated against baseline data. According to guideline criteria, PPF was diagnosed if at least two of the following criteria were met: worsening of respiratory symptoms, physiological evidence of progression, or radiological evidence of progression. Physiological evidence of progression was defined as either a 5% decrease in FVC or a 10% decrease in DLCO within 1 year. Radiological evidence of progression was defined as the progression of radiological findings within a 1-year period (17).

Basic information included: gender, age, body mass index, smoking history, and pulmonary arterial hypertension. Clinical manifestations included: fever, cough, chest tightness, shortness of breath, muscle weakness, arthritis, Heliotrope rash, mechanic’s hands, Gottron’s sign, periungual capillary change.

Myositis antibodies were categorized into myositis-specific antibodies (MSA) and myositis-associated antibodies (MAA). In brief, we collected serum samples at baseline, before initiation or escalation of immunosuppressive therapy. MSA and MAA were measured using a commercial Euroimmun line blot assay, performed according to the manufacturer’s instructions. Results were classified as positive or negative according to the manufacturer-defined cutoff. Weakly positive results were confirmed by enzyme-linked immunosorbent assay (ELISA) (Medical & Biological Laboratories, Beijing Biotech Co.). Laboratory tests included complete blood count, albumin, muscle enzyme levels, C-reactive protein, erythrocyte sedimentation rate, ferritin, common inflammatory factors, and lymphocyte subsets.

Imaging examinations included HRCT and ^18^F-FDG PET-CT. Based on HRCT findings, patients were classified into NSIP, UIP, NSIP+OP, and OP. Patients fasted for ≥6 h, had blood glucose level <7.8mmol/L, and imaging was performed 60 min after injection ^18^F-FDG (3.7 MBq/Kg). Measurements were performed independently by two nuclear medicine physicians with more than 10 years of experience. A fixed 20-mm-diameter region of interest (ROI) was placed over the area of maximal pulmonary FDG uptake in the bilateral lung parenchyma on axial slices from the lung apex to the diaphragm. The standardized uptake value (SUV) was calculated using the following formula: SUV (g/mL) = regional radioactivity concentration (Bq/ml)/[injected dose (Bq)/body weight (g)]. The lung parenchyma within each ROI was evaluated on HRCT for ground-glass attenuation, consolidation, and reticulation. The ROI excluded the mediastinum, hilar vessels, pleura, and obvious non-parenchymal uptake. The ^18^F-FDG PET-CT included the SUV_max_ for both lungs, mediastinal lymph nodes, hilar lymph nodes, and muscles.

Pulmonary function tests included FVC%pred and DLCO%pred. Treatment included immunomodulators, Janus kinase (JAK) inhibitors, immunosuppressants, antifibrotic drugs, rituximab, corticosteroids, and the dosage of initial corticosteroids.

### Statistical analysis

Statistical analysis were performed using SPSS 26.0, GraphPad Prism 9.0 and R software (4.4.1). Continuous variables were expressed as mean ± standard deviation ( $\bar{X}$± *s*) or median with interquartile range (IQR), depending on their distribution characteristics. If the data met the normal distribution assumption, they were analyzed using the *t*-test; otherwise, the Mann-Whitney *U* test was used. Categorical variables were expressed as relative frequencies, and intergroup comparisons were conducted using the *χ²* test. Univariate and multivariate analyses were performed using logistic regression analysis. Variables showing univariate association (*P* < 0.05) with PPF were selected for a multivariate logistic regression. A nomogram was then developed based on these multivariate results (*P* < 0.05) to build predictive models. Calibration curves, generated via the Kaplan–Meier method, assessed agreement between nomogram predictions and actual observations. The Hosmer–Lemeshow test was employed to assess model calibration, with a *P*-value >0.05 indicating a good fit. The nomogram’s performance was compared against each factor using area under the curve (AUC) derived from receiver operating characteristic (ROC) curves. To determine clinical utility, decision curve analysis (DCA) was performed by quantifying net benefits for PPF across various threshold probabilities. The survival rate was calculated using the Kaplan-Meier curve. A *P* value <0.05 was considered statistically significant.

### Results

From January 2018 to December 2024, our hospital admitted a total of 271 patients with IIM-ILD, of which 71 patients met the inclusion and exclusion criteria (**Figure 1**). Among the included patients, 29 patients (40.8%) developed PPF. The median time from IIM-ILD diagnosis to PPF was 61 days (IQR 25–161), compared with 237.5 days (IQR 161–378.5) for the non-PPF group (*P* < 0.001). **Figure 2** presents two cases: one case with baseline ^18^F-FDG PET-CT showing high SUV_max_ in bilateral lungs, which progressed to PPF within the following year; and another case with mild metabolic elevation in bilateral lungs on baseline ^18^F-FDG PET-CT, which remained stable during the subsequent year of follow-up.

**Figure 1:**
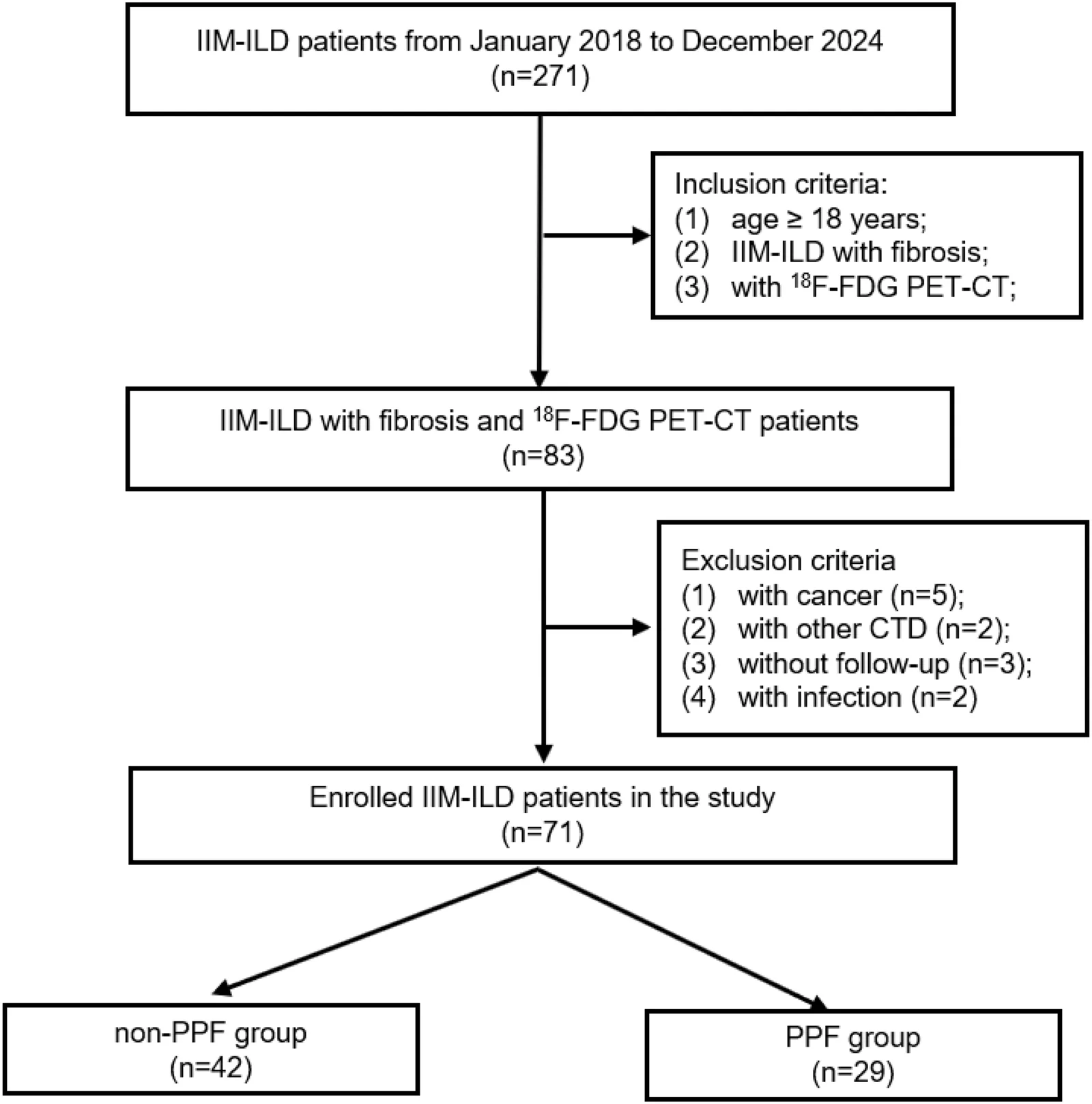
Study selection process flowchart.

**Figure 2:**
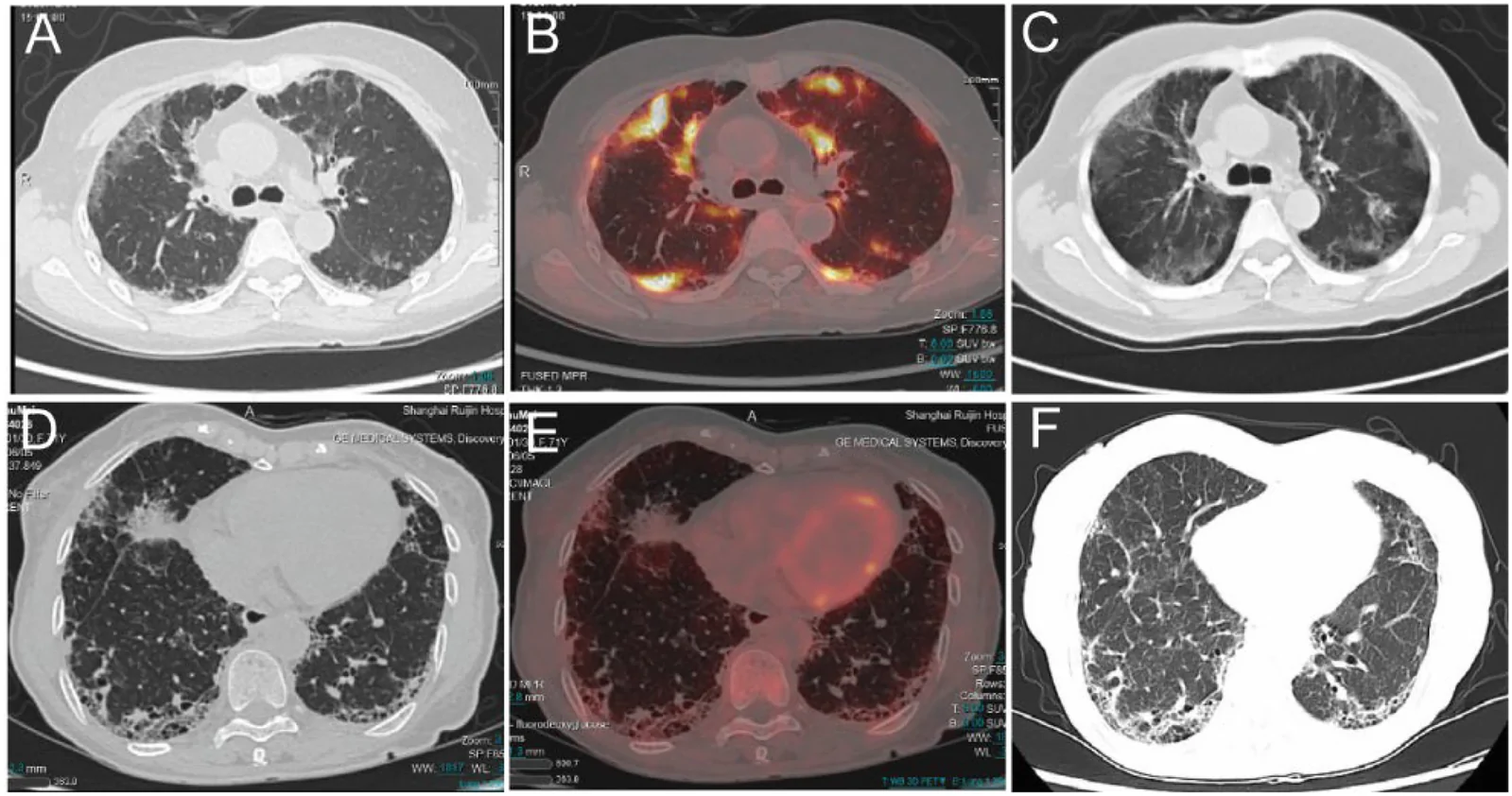
**(A)** The baseline HRCT of a PPF case; **(B)** the PPF case with high bilateral lungs SUV in baseline ^18^F-FDG PET-CT; **(C)** the follow up HRCT of a PPF case; **(D)** the baseline HRCT of a stable case; **(E)** the stable case with low bilateral lungs SUV in baseline ^18^F-FDG PET-CT; **(F)** the follow up HRCT of a stable case.

In terms of clinical characteristics, there were no significant differences between the two groups regarding gender, age, body mass index, smoking history, an presence of pulmonary arterial hypertension. The proportion of DM in the PPF group was significantly higher than in the non-PPF group (69.0% vs 42.9%, *P* = 0.030), while the proportion of ASS was lower in the PPF group (27.6% vs 50.0%, *P* = 0.059); the proportions of other types of myositis were similar. Regarding clinical manifestations, the proportion of patients presenting with cough symptoms was significantly lower in the PPF group, while the proportions of Heliotrope rash and Gottron’s sign were significantly higher in the PPF group compared to the non-PPF group (all *P* < 0.05). In terms of MSA/MAA, the proportion of anti-MDA5 antibody positivity (65.5% vs 31.0%, *P* = 0.004) and the proportion of anti-Ro52 antibody positivity (93.1% vs 64.3%, *P* = 0.005) were significantly higher in the PPF group; the positivity rates of antisynthetase antibodies were lower in the PPF group but did not reach statistical significance. Laboratory findings revealed that neutrophil-to-lymphocyte ratio (NLR), C-reactive protein, ferritin, and lactate dehydrogenase (LDH) levels were significantly higher in the PPF group compared to the non-PPF group (all *P* < 0.05). Conversely, albumin levels, CD4^+^ T cell counts, CD3^+^CD4^+^ T cell counts, and CD19^+^ B cell counts were significantly lower in the PPF group (all *P* < 0.05). Regarding imaging classification, NSIP was the main ILD subtype in both groups. In ^18^F-FDG PET-CT, the SUV_max_ for both lungs was significantly higher in the PPF group (*P* = 0.001) (**Figure 3A**), and the SUV_max_ for mediastinal and hilar lymph nodes was also slightly higher in the PPF group (*P* = 0.073, 0.054). Concerning pulmonary function, FVC% pred was significantly lower in the PPF group compared to the non-PPF group (*P* = 0.020) (**Supplementary Figure S1A**), and DLCO% pred was slightly lower in the PPF group (*P* = 0.051). In terms of treatment, the dosage of initial corticosteroids was higher in the PPF group but did not reach significance (*P* = 0.164). The proportion of patients using antifibrotic drugs (nintedanib, pirfenidone) was slightly higher in the PPF group (41.4% vs 35.7%, *P* = 0.629), with no significant differences observed in the use of JAK inhibitors and immunosuppressants (**Table 1**).

**Figure 3:**
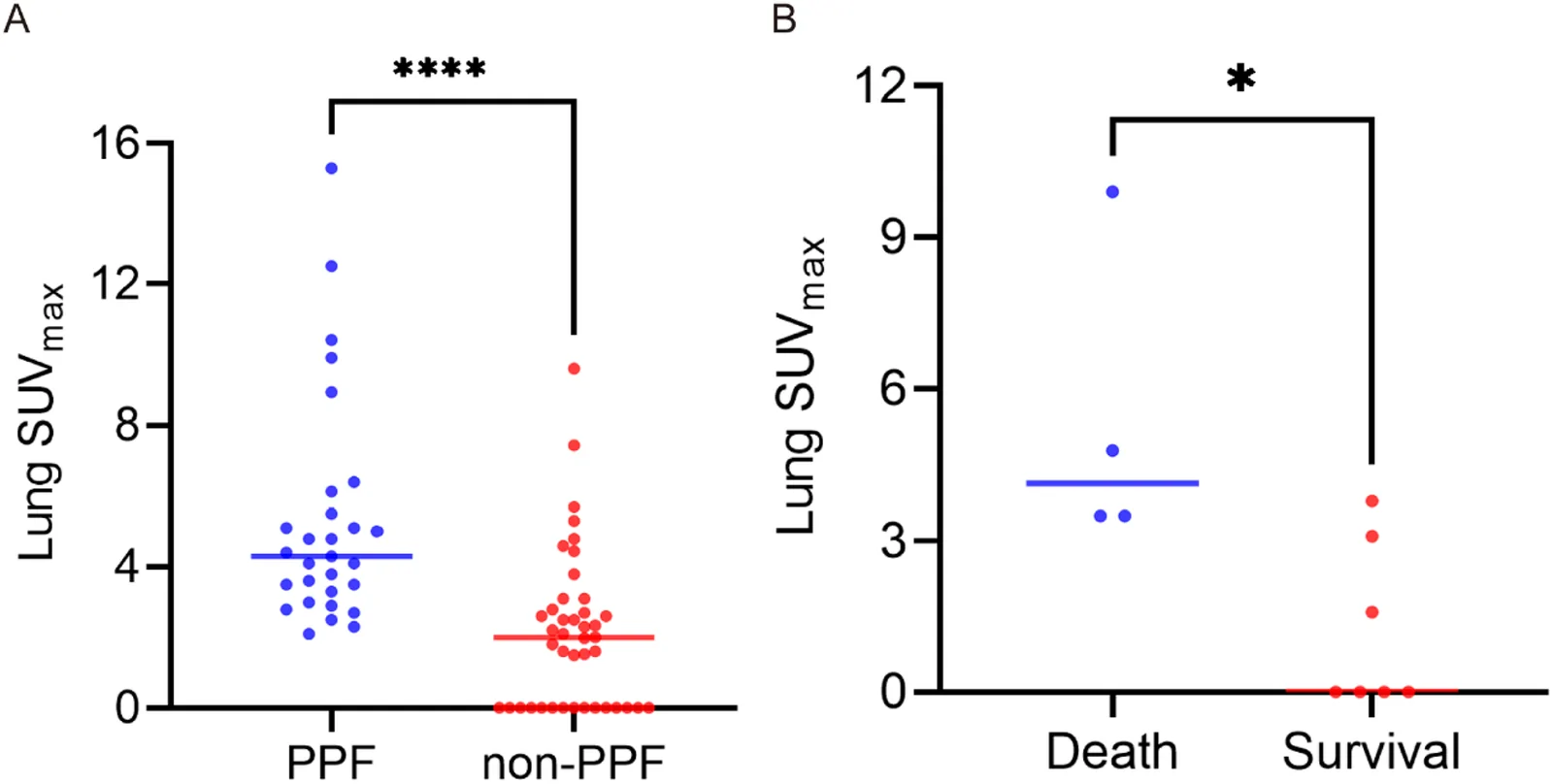
**(A)** The scatter plot of lung SUV_max_ comparsion between PPF and non-PPF; **(B)** The scatter plot of lung SUV_max_ comparsion between patients who died within one year and those who remained alive for more than five years.

**Table 1:** Comparisons of multiple factors between non-PPF and PPF groups.

Variables	non-PPF (n=42)	PPF (n=29)	*P* value
Demographics			
Male (%)	10 (23.8)	7 (24.1)	0.975
Age (year)	55.86 ± 11.77	55.38 ± 13.56	0.875
BMI (kg/m^2^)	22.43 ± 3.27	23.58 ± 2.97	0.134
Smoking history (%)	7 (16.7)	3 (10.3)	0.685
Pulmonary hypertension (%)	4 (9.5)	5 (17.2)	0.550
IIM clinical phenotype (%)			
ASS	21 (50.0)	8 (27.6)	0.059
Dermatomyositis	18 (42.9)	20 (69.0)	0.030
Polymyositis	1 (2.4)	0 (0.0)	0.410
IMNM	2 (4.8)	1 (3.4)	0.790
Clinical features (%)			
Fever	9 (21.4)	6 (20.7)	0.940
Cough	25 (59.5)	10 (34.5)	0.038
Chest tightness	16 (38.1)	13 (44.8)	0.571
Shortness of breath	20 (47.6)	16 (55.2)	0.531
Muscle weakness	13 (31.0)	14 (48.3)	0.139
Arthritis	19 (45.2)	12 (41.4)	0.747
Heliotrope rash	3 (7.1)	14 (48.3%)	<0.001
Mechanic’s hands	14 (33.3)	9 (31.0)	0.839
Gottron’s sign	9 (21.4)	13 (44.8)	0.036
Periungual capillary changes	6 (14.3)	7 (24.1)	0.291
Myositis antibodies (%)			
Anti-Mi-2	6 (14.3)	0 (0.0)	0.090
Anti-MDA5	13 (31.0)	19 (65.5)	0.004
Anti-JO-1	8 (19.0)	4 (13.8)	0.796
Anti-PL-7	6 (14.3)	1 (3.4)	0.271
Anti-PL-12	6 (14.3)	1 (3.4)	0.271
Anti-EJ	8 (19.0)	4 (13.8)	0.796
Anti-Ro52	27 (64.3)	26 (93.1)	0.005
Laboratory finding			
NLR	3.32 (2.33,6.56)	6.37 (3.44, 8.68)	0.015
CRP (mg/mL)	2.00 (1.00,10.23)	6.00 (2.50, 25.50)	0.008
ESR (mm/L)	17.00 (6.75, 25.25)	21.00 (13.00, 42.00)	0.062
Ferritin (ng/mL)	272.75 (76.48, 331.93)	622.55 (198.70, 1415.15)	0.003
CA-125 (U/mL)	12.75 (7.90-19.80)	14.60 (7.90-20.40)	0.712
Interleukin-6 (pg/mL)	12.37 (5.92, 27.30)	16.50 (5.00, 27.80)	0.572
Albumin (g/L)	36.50 (32.00, 40.00)	31.00 (29.00, 35.00)	0.001
Creatine kinase (U/L)	89.00 (35.75, 181.50)	98.00 (54.00, 540.00)	0.164
LDH (U/L)	248.5 (211.25-291.75)	326.0 (256.00-390.00)	<0.001
CD3^+^ T cell	902.00 (578.50, 1003.00)	731.00 (458.00, 833.50)	0.028
CD3^+^CD4^+^ T cell	608.00 (364.00, 685.50)	469.00 (289.50, 525.50)	0.029
CD3^+^CD8^+^ T cell	271.00 (151.50, 308.00)	213.00 (104.50, 251.00)	0.097
NK cell	152.00 (61.50, 167.00)	115.00 (51.00, 135.50)	0.156
CD19^+^ B cell	219.00 (137.00, 322.50)	139.50 (83.75, 169.25)	0.002
Radiographic pattern (%)			
NSIP	34 (81.0)	25 (86.2)	0.796
UIP	4 (9.5)	0 (0.0)	0.235
NSIP+OP	1 (2.4)	3 (10.3)	0.364
OP	3 (7.1)	1 (3.4)	0.889
^18^F-FDG PET/CT (SUV_max_)			
Bilateral lungs	1.99 (0.00, 2.88)	4.30 (3.15, 5.82)	<0.001
Mediastinal lymph nodes	0.00 (0.00, 3.10)	0.00 (0.00, 4.90)	0.073
Hilar lymph nodes	0.00 (0.00, 0.10)	0.00 (0.00, 4.58)	0.054
Muscle	0.00 (0.00, 2.55)	0.00 (0.00, 3.57)	0.382
Pulmonary function tests			
FVC% pred	79.55 (61.70, 97.63)	65.00 (46.70, 75.40)	0.020
DLCO% pred	71.30 (64.48, 81.18)	65.30 (50.90, 73.50)	0.051
Treatment			
Immunomodulator (%)	20 (47.6)	13 (44.8)	0.817
JAK inhibitor (%)	3 (7.1)	5 (17.2)	0.347
Immunosuppressant (%)	30 (71.4)	17 (58.6)	0.262
Anti fibrotic drugs (%)	15 (35.7)	12 (41.4)	0.629
Rituximab (%)	6 (14.3)	5 (17.2)	0.996
Corticosteroids (%)	40 (95.2)	29 (100.00)	0.510
Initial corticosteroids dosage (mg)	50.00 (37.5, 60.00)	100.00 (60.00, 100.00)	0.164

PPF, progressive pulmonary fibrosis; BMI, body mass index; ASS, antisynthetase syndrome; IMNM: Immune-mediated necrotizing myopathy;.

NLR, neutrophil-to-lymphocyte ratio; CRP, C-reactive protein; ESR, erythrocyte sedimentation rate; LDH, lactate dehydrogenase; CA-125, carbohydrate antigen 125; NSIP non-specific interstitial pneumonia; UIP, usual interstitial pneumonia; OP, organizing pneumonia; FVC%, percent-predicted forced vital capacity; DLCO, diffusion capacity of the lungs for carbon monoxide; JAK, Janus kinase.

In the univariate logistic regression analysis, we found that PPF was associated with DM, cough, Heliotrope rash, Gottron’s sign, anti-MDA5 antibody positivity, anti-Ro52 antibody positivity, NLR, albumin, ferritin, CD19^+^ B cell counts, bilateral lungs SUV_max_, and FVC% pred (all *P* < 0.05) (**Table 2**). In further multivariate analysis, bilateral lungs SUV_max_ (*OR* = 3.600, 95%*CI*: 1.470-8.816, *P* = 0.005) and FVC% pred (*OR* = 0.923, 95%*CI*: 0.816-0.989, *P* = 0.022) were associated with the occurrence of PPF (**Table 2**).

**Table 2:** Risk factors for predicting PPF using univariate and multivariate analysis.

Regression analysis	*OR*	95%*CI*	*P*
Univariate analysis			
Dermatomyositis	3.268	1.203-8.877	0.023
Cough	0.358	0.134-0.956	0.040
Heliotrope rash	12.133	3.047-48.317	<0.001
Gottron’s sign	2.979	1.054-8.417	0.039
Anti-MDA5	4.238	1.548-11.604	0.005
Anti-Ro52	1.860	1.097-3.154	0.021
NLR	1.182	1.023-1.367	0.023
Albumin	0.853	0.762-0.954	0.005
LDH	1.008	1.003-1.014	0.007
Ferritin	1.001	1.000-1.003	0.010
CD19^+^ B cell counts	0.993	0.988-0.998	0.005
Lung SUV_max_	1.723	1.272-2.334	<0.001
FVC% pred	0.965	0.934-0.997	0.032
Anti fibrotic drugs	1.271	0.481-3.358	0.629
Initial corticosteroids dosage	1.012	0.999-1.024	0.071
Multivariate analysis			
Lung SUV_max_	3.600	1.470-8.816	0.005
FVC% pred	0.923	0.861-0.989	0.022

*OR*, odds risk; *CI*, confidence interval; NLR, neutrophil-to-lymphocyte ratio; LDH, lactate dehydrogenase; FVC%pred, percent-predicted forced vital capacity.

Using multivariable analysis, a nomogram was developed to derive each selected patient’s total score and PPF probability. Meanwhile, the score and probability of a PPF patient are illustrated in the nomogram (**Figure 4**).

**Figure 4:**
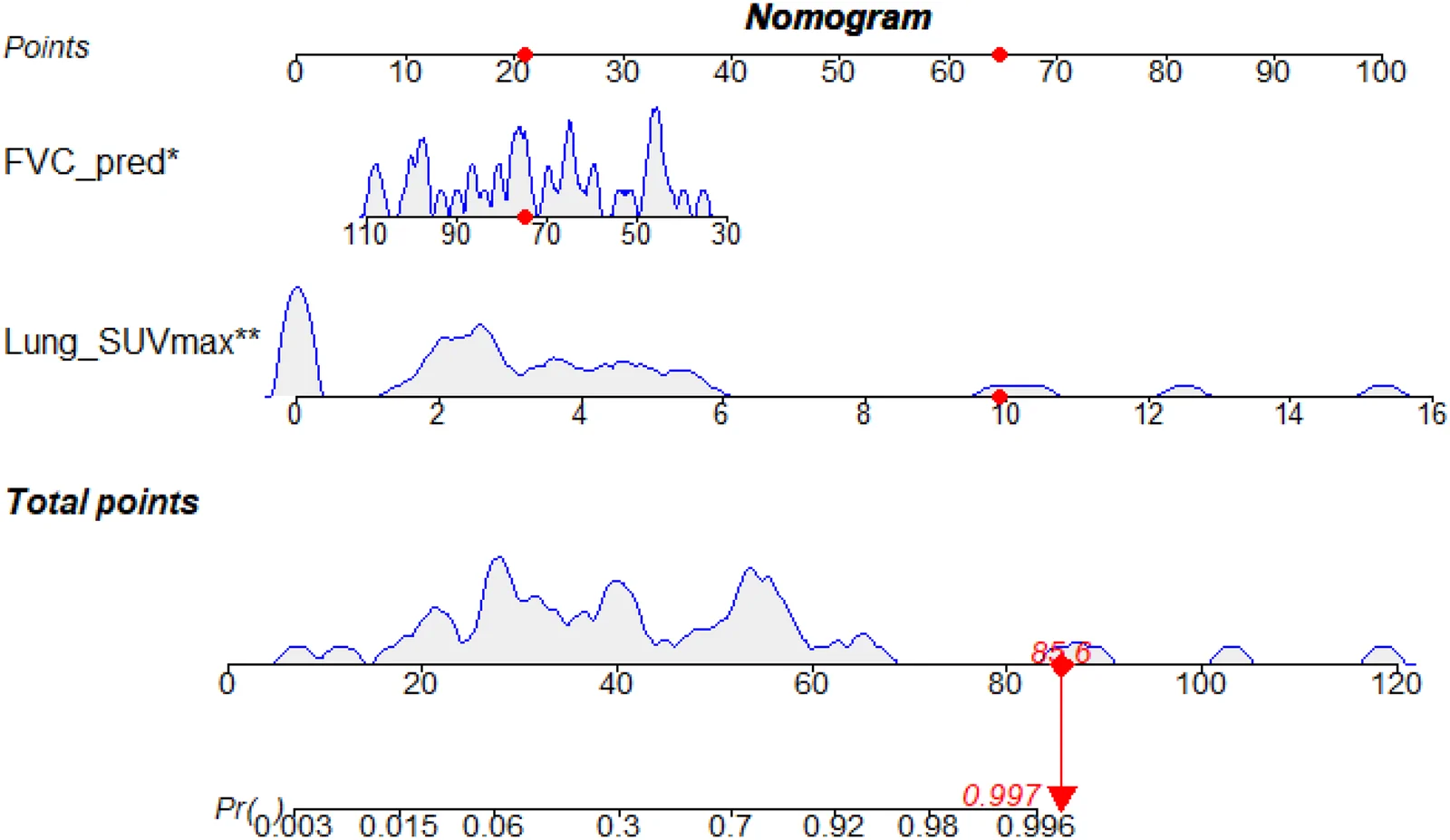
Nomogram derived from multivariable analysis for predicting PPF.

Calibration plots revealed strong agreement between nomogram predictions and actual observations (**Figure 5A**). Results from the Hosmer–Lemeshow test indicated a good fit for the nomogram (*P* = 0.143). In ROC curve analysis, the cut-off value for bilateral lungs SUV_max_ was >2.65 and an AUC of 0.844 (95%*CI*: 0.753-0.934). For FVC% pred, the cut-off was <76.65% and an AUC of 0.783 (95%*CI*: 0.666-0.901). The occurrence of PPF was significantly higher in patients with lung SUVmax >2.65 (68.4% vs 9.1%, *P* < 0.001) and in those with FVC%pred <76.65% (61.5% vs 15.8%, *P* < 0.001). When combining two significant factors, the AUC for predicting PPF reached 0.887 (95%*CI*: 0.805-0.969) (**Figure 5B**). DCA demonstrated that the combined model offered superior clinical benefit and performance compared to any single factor (**Figure 5C**).

**Figure 5:**
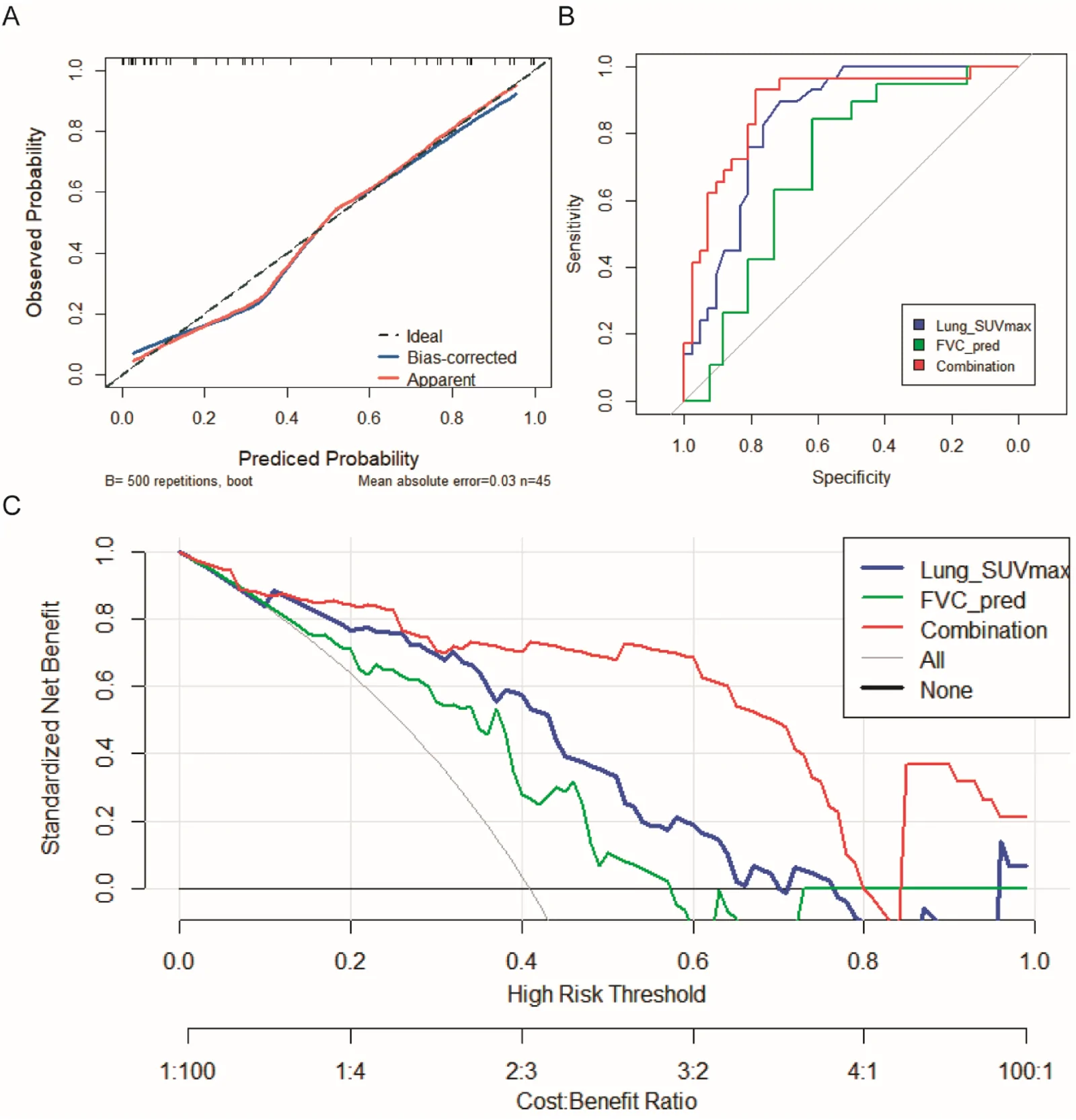
**(A)** The calibration curve of the combination prediction model; **(B)** ROC curve of lung SUV_max_, FVC% pred, and combination model predicting PPF; **(C)** the decision curve analysis of lung SUV_max_, FVC% pred, and combination model.

During the follow-up, five deaths occurred in the PPF group, while only one death was observed in the non-PPF group. Notably, four of the five deaths in the PPF group occurred within the first year. To further investigate the association between lung SUVmax and mortality, we compared lung SUV_max_ between patients who died within one year and those who survived for more than five years. Lung SUV_max_ was significantly higher in patients who died within one year (**Figure 3B**). However, the decreased FVC%pred was not associated with short-term mortality (**Supplementary Figure S1B**). The Kaplan-Meier survival analysis demonstrated that the survival rate of patients without PPF was significantly higher than that of patients with PPF (*P* = 0.04, **Figure 6**).

**Figure 6:**
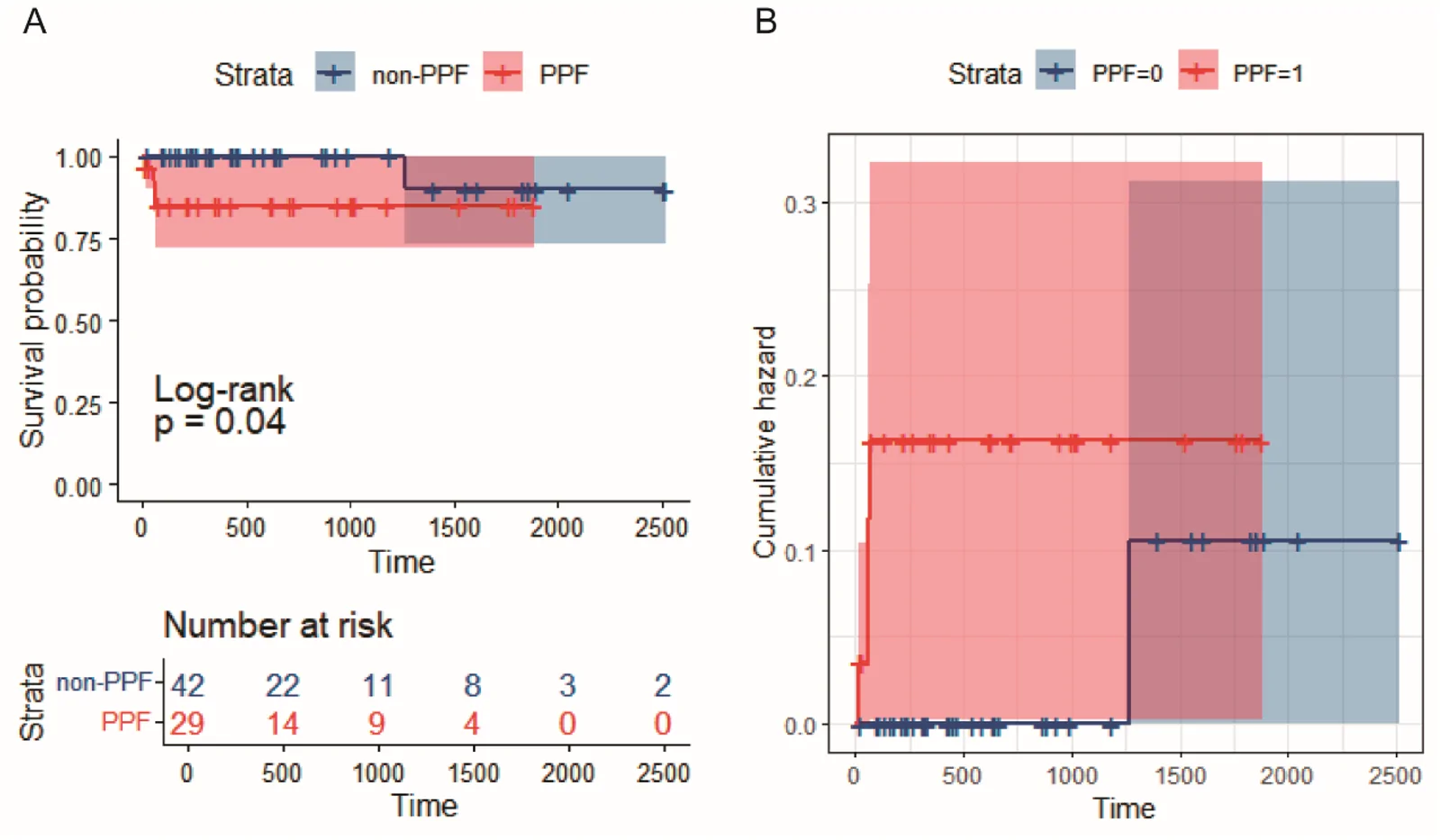
**(A)** The survival curve of PPF and non-PPF, as well as their risk table; **(B)** the cumulative event curve of PPF and non-PPF.

## Discussion

The median time to PPF diagnosis in our cohort was shorter than that observed in patients without PPF, suggesting that the progression of pulmonary fibrosis in our cohort may be associated with cumulative chronic damage and poor response to immunosuppressive therapy.

^18^F-FDG PET-CT has previously been utilized for screening of malignancies in patients with IIM, and it can also be used to determine the type of ILD. Previous studies have demonstrated that ^18^F-FDG PET-CT has potential value in predicting RP-ILD (6, 11). Moreover, research indicated that high FDG uptake was associated with active ILD in connective tissue diseases (18). To explore the predictive value of ^18^F-FDG PET-CT in PPF, we compared the SUV_max_ of the bilateral lungs, mediastinal lymph nodes, hilar lymph nodes, and muscles between PPF and non-PPF groups. It was observed that the bilateral lung SUV_max_ in the PPF group was significantly higher than in the non-PPF group. Further univariate and multivariate analysis revealed that high lung SUV_max_ and decreased FVC% pred were risk factors for the occurrence of PPF. Later, we constructed and validated a nomogram model for predicting the risk of PPF in IIM-ILD patients. Lastly, we found that the prognosis of PPF was poor compared with non-PPF.

Animal experiments have shown that bleomycin-induced mouse models of pulmonary fibrosis exhibit increased ^18^F-FDG uptake, suggesting that ^18^F-FDG PET-CT may non-invasively assess the degree of pulmonary fibrosis (19). The progression of pulmonary fibrosis is often accompanied by inflammatory responses and fibroblast activation. High FDG uptake may reflect locally metabolically active inflammation or fibrotic areas, providing a basis for early identification of disease progression. Studies have found that in UIP patients, the expression level of the neovascular marker CD105 correlates with lung FDG uptake and prognosis, indicating a potential role of microvasculature in ILD (20). Our study demonstrated that lung SUV_max_ served as a predictor for IIM-ILD progression and was associated with short-term mortality in PPF patients.

Decreased pulmonary function is one of the core characteristics of PPF, which may interact with the condition. A previous study showed that poor pulmonary function test results were associated with the occurrence of PPF in fibrosis-ILD (21). A FVC decline of 5% within 12 months was associated with an approximately 2-fold increase in mortality compared to stable FVC (22). A relative decline in FVC of approximately 10% was the strongest predictor of reduced transplant-free survival (23). Our study found that decreased FVC% pred was correlated with the occurrence of PPF, which was consistent with previous findings.

Previous studies have reported that LDH and CA-125 have predictive value in anti-PL7-positive ASS (24), which contrasts with our findings. This discrepancy may be because anti-PL7-positive patients represented only a small subset of our IIM cohort and because the predictive value of these markers may be attenuated when PET-CT-derived parameters are included in the model.

In this study, we specifically evaluate the predictive and prognostic value of ^18^F-FDG PET-CT parameters for PPF in IIM-ILD patients. While FAPi PET/CT has recently been explored for imaging fibroblast activation in IIM-ILD (15, 16), our study addresses a distinct biological pathway (glucose metabolism) and a separate clinical question (PPF prediction and prognosis) via the widely accessible ^18^F-FDG tracer. Firstly, our study found that increased bilateral lung SUV_max_ correlates with the occurrence of PPF. Secondly, we found that increased bilateral lung SUV_max_ was associated with the short-term mortality of PPF. Finally, combined analysis of risk factors showed good performance in predicting PPF, offering clinical decision-making value.

Based on our findings, we hypothesize that normalization or significant reduction of pulmonary FDG uptake on follow-up PET-CT is associated with effective suppression of inflammation activation, a lower risk of progressive fibrosis, and a better prognosis. However, confirming whether PET normalization can translate into clinical efficacy will require further well-designed prospective longitudinal studies.

Our study also has several limitations. Firstly, given the small sample size and the preliminary, exploratory nature of this study, the combined predictive value could not be further externally validated. Future multicenter prospective cohort studies are therefore needed to validate our findings. Secondly, Krebs von den Lungen-6, a marker related to pulmonary fibrosis, was not tested in patients a few years ago, thus not included in this study. Thirdly, baseline HRCT scans were not systematically scored for GGO extent, precluding evaluation of this established prognostic factor. The absence of GGO quantification may have introduced confounding, as GGO burden could have influenced both PET-CT uptake patterns and PPF outcomes. Future prospective studies incorporating both PET-CT parameters and quantitative HRCT metrics—ideally using automated deep learning approaches as employed by Qiang et al.—are needed to determine whether PET-CT provides incremental predictive value beyond the information already captured by GGO and other HRCT features (25). Fourthly, our findings may not be fully generalizable to ambulatory patients with mild or stable disease, which may introduce a selection bias. Fifthly, SUV_max_ may not fully capture the diffuse and density-dependent nature of FDG uptake in IIM-ILD. Alternative parameters such as SUV_mean_, density-corrected SUV, total lung glycolysis, and percent high-uptake lung volume may offer more physiologically meaningful quantification of pulmonary metabolic activity in this context. Future prospective studies incorporating these volumetric and density-corrected parameters—ideally alongside the PET visual scoring approach used by Zhang et al.—are needed to determine which PET-CT–derived metric provides the greatest incremental predictive value for PPF in IIM-ILD (12). Lastly, different types of IIM with ILD might have varying disease progressions, such as MDA5-positive DM and ASS, but due to the small sample size, subgroup analysis was not suitable. Despite these limitations, our study confirmed that ^18^F-FDG PET-CT correlated with the development and prognosis of PPF in IIM-ILD, and a visualized nomogram can effectively predict the onset of PPF.

## Conclusions

^18^F-FDG PET-CT and FVC%pred are associated with the occurrence of PPF in patients with IIM-ILD. The prognosis of PPF is poor, and high SUV_max_ is correlated with short-term mortality. Exhibiting robust discrimination and calibration, this nomogram holds potential clinical value in predicting the development and death from PPF in patients with IIM-ILD.

## Data Availability

The datasets presented in this article are not readily available because of privacy concerns. Requests to access the datasets should be directed to Chao sun, chaosunrj@163.com.
