# Supplementary material for: The role of 18F-FDG PET-CT in predicting progressive pulmonary fibrosis in idiopathic inflammatory myopathy associated interstitial lung disease
Source: Front Immunol. 2026 Oct 29;17:1862578. doi: 10.3389/fimmu.2026.1862578 (PMC13754872; doi:10.3389/fimmu.2026.1862578)
Supplement: Supplementary file 1 [file DataSheet1.docx]

The role of ^18^F-FDG PET-CT in predicting progressive pulmonary fibrosis in idiopathic inflammatory myopathy associated interstitial lung disease

**Fig. S1** (A)The scatter plot of lung FVC% pred comparison between PPF and non-PPF. (B) The scatter plot of lung FVC% pred comparison between patients who died within one year and those who remained alive for more than five years
